# Early Duodenal Perforation Due to Migrated Plastic Biliary Stent, Successfully Managed Endoscopically: A Case Report

**DOI:** 10.1155/crgm/1965411

**Published:** 2026-08-20

**Authors:** Tamzyn Huisamen, Shiraz Gabriel, Ahmed Abdelsalam, Renaldo Kortje, Desiree Moodley

**Affiliations:** ^1^ Division of Gastroenterology, Department of Medicine, Faculty of Medicine and Health Sciences, Stellenbosch University and Tygerberg Hospital, Cape Town, South Africa, sun.ac.za; ^2^ Department of Surgery, Faculty of Medicine and Health Sciences, Stellenbosch University and Tygerberg Hospital, Cape Town, South Africa, sun.ac.za

**Keywords:** biliary stent, duodenal perforation

## Abstract

Endoscopic retrograde cholangiopancreatography (ERCP) with plastic biliary stent placement is a common treatment for biliary obstruction. Although stent migration occurs in up to 10% of cases, duodenal perforation is rare and associated with significant morbidity if diagnosis is delayed. We report the case of a man in his 60s who underwent ERCP with insertion of a 70‐mm duodenal‐bend plastic biliary stent for acute cholangitis. Ten days later, he re‐presented with acute abdominal pain and elevated inflammatory markers. Imaging and repeat ERCP demonstrated distal stent migration with duodenal perforation. The stent was removed, and the defect was successfully managed with a single through‐the‐scope clip. Notably, this complication occurred despite the absence of recognised risk factors for stent migration or perforation, suggesting that clinically significant stent‐related complications may occur even in patients considered to be at relatively low risk. This case emphasises the need to consider stent‐related complications in any patient presenting with abdominal pain after ERCP and demonstrates that early endoscopic management can achieve favourable outcomes in appropriately selected patients.

## 1. Introduction

Endoscopic retrograde cholangiopancreatography (ERCP) is a widely utilised minimally invasive technique for the management of biliary tract obstruction, with plastic stents commonly used to facilitate endoscopic biliary drainage [1]. Stent migration is a recognised adverse event associated with plastic biliary stent placement, occurring in approximately 5%–10% of cases, whereas perforation is rare, accounting for less than 1% of reported complications [2, 3].

Only a small number of case reports have described duodenal perforation caused by early biliary stent migration, and most involve delayed presentation or have required surgical intervention. Identified risk factors include the use of longer stents (> 120 mm), straight stent configuration, and retention of the proximal portion of the stent within the common bile duct (CBD) [3, 4].

In this report, we describe a case of duodenal perforation following migration of a 70‐mm duodenal‐bend plastic stent, which was successfully managed endoscopically using a single through‐the‐scope clip. Although uncommon, duodenal perforation is associated with significant morbidity, particularly when diagnosis and treatment are delayed. In contrast to most published cases, perforation occurred within 10 days of stent placement despite the absence of recognised risk factors for stent migration or duodenal perforation. This case suggests that serious stent‐related complications may still occur in patients who do not fit the conventional high‐risk profile and supports maintaining clinical suspicion when patients develop abdominal pain after ERCP. It also illustrates that early endoscopic intervention can be an effective alternative to surgery in appropriately selected patients.

## 2. Case

A 61‐year‐old male presented to the emergency department on 4 December 2025 with an acute onset of fever, vomiting and right upper quadrant abdominal pain. These symptoms were preceded by a 3‐week history of painless jaundice and generalised pruritus.

Initial laboratory investigations demonstrated a mixed pattern of liver injury with cholestatic predominance, with a total bilirubin of 525 μmol/L (conjugated bilirubin 487 μmol/L), alkaline phosphatase (ALP) 315 U/L, gamma‐glutamyl transferase (GGT) 498 U/L, alanine aminotransferase (ALT) 268 U/L and aspartate aminotransferase (AST) 207 U/L. Inflammatory markers were significantly elevated, with a C‐reactive protein (CRP) of 52 mg/L and a white cell count of 24.10 × 10^9^/L. (Table 1)

**TABLE 1:** Laboratory results.

Variable	Reference range	Pre‐ERCP	Post‐ERCP	Readmission
Total bilirubin (μmol/L)	5–21	525	259	109
Conjugated bilirubin (μmol/L)	0–3	487	239	109
Alanine transaminase (ALT) (U/L)	10–40	268	447	75
Aspartate transaminase (AST) (U/L)	15–40	207	501	142
Alkaline phosphatase (ALP) (U/L)	53–128	315	345	518
Gamma‐glutamyl transferase (GGT) (U/L)	< 68	498	415	581
International normalised ratio (INR)		1.26		1.25
C‐reactive protein (CRP) (mg/L)	< 10	52		310
White cell count (x10^9^/L)	3.92–10.40	24.10		9.87

Contrast‐enhanced abdominal computed tomography (CT) revealed cholelithiasis and choledocholithiasis with marked dilatation of the CBD, as well as intrahepatic and extrahepatic biliary dilatation. Additionally, multiple hypodense, heterogeneous lesions were identified within the liver parenchyma.

**FIGURE 1:**
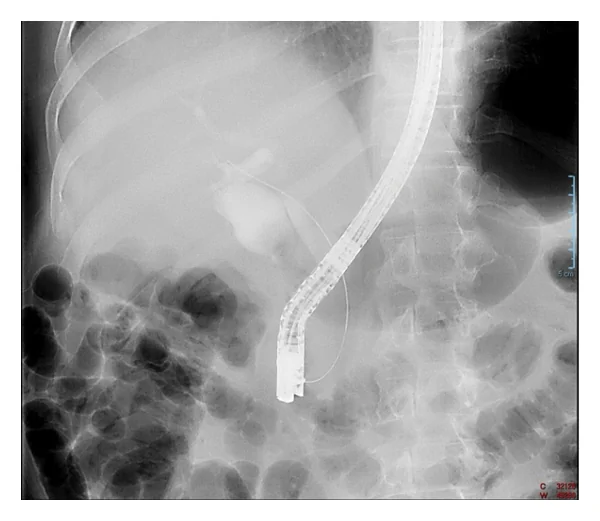
Fluoroscopic view demonstrating a dilated biliary tree with a large filling defect within the common bile duct.

The patient was subsequently diagnosed with ascending cholangitis secondary to choledocholithiasis, complicated by multiple liver abscesses. ERCP performed on the day of presentation demonstrated a markedly dilated biliary tree with a large filling defect within the CBD (Figure 1). Given the patient’s critical clinical condition, a 10‐French, 70‐mm duodenal‐bend plastic biliary stent (Advanix Biliary, Boston Scientific, USA) was inserted, with appropriate positioning confirmed fluoroscopically during the procedure. The liver abscesses were managed with ultrasound‐guided pigtail catheter drainage. Following treatment, the patient’s clinical condition and liver biochemistry improved, and he was discharged with plans for interval ERCP in 6 weeks.

On 14 December 2025, 10 days after the index ERCP, the patient re‐presented with diffuse abdominal pain associated with nausea and vomiting. On assessment in the emergency department, he was noted to have a temperature of 37.4°C, a blood pressure of 123/71 mmHg and a pulse rate of 93 beats per minute. He had generalised abdominal tenderness without clinical evidence of peritonitis. Repeat blood tests demonstrated a marked inflammatory response, with a CRP of 310 mg/L. (Table 1)

Repeat abdominal CT findings were suggestive of duodenal perforation with possible choledochoduodenal fistulous communication. In the absence of free intraperitoneal air and given the patient’s haemodynamic stability, a contained retroperitoneal perforation was suspected.

**FIGURE 2:**
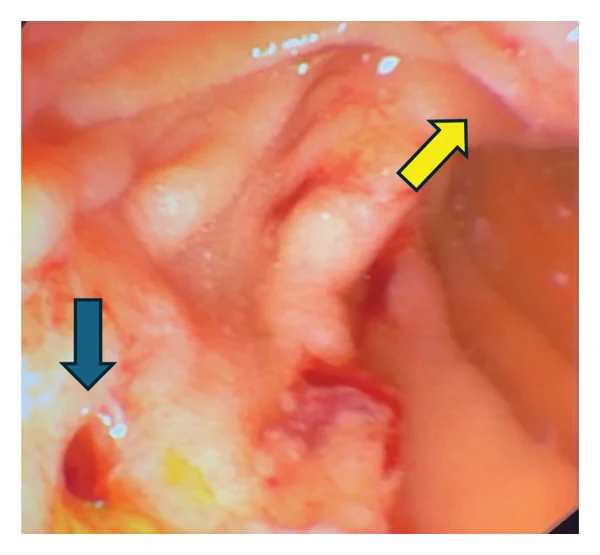
Endoscopic view showing perforation of the lateral duodenal wall (blue arrow) opposing the major duodenal papilla (yellow arrow).

**FIGURE 3:**
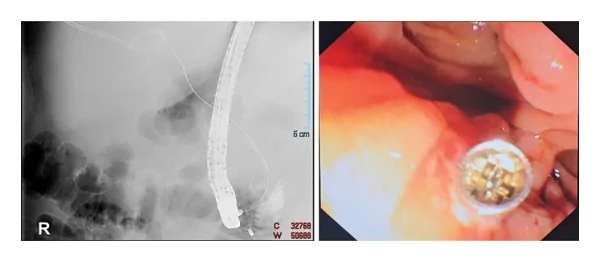
Fluoroscopic (left) and endoscopic (right) views showing closure of duodenal defect with a single through‐the‐scope clip.

A repeat ERCP was performed on 17 December 2025. Endoscopy demonstrated distal migration of the biliary stent, with the distal tip perforating the lateral wall of the second part of the duodenum, resulting in a well‐circumscribed 5 × 5 mm transmural defect (Figure 2). There was no evidence of active contamination. The migrated stent was removed endoscopically, and the perforation was successfully closed using a single through‐the‐scope clip (Figure 3). Cholangiography demonstrated a patent biliary tree with spontaneous passage of the previously identified CBD stone and no residual biliary obstruction. However, given the recent severe episode of ascending cholangitis and the need to maintain biliary drainage during recovery, a new plastic biliary stent was inserted, with satisfactory biliary drainage observed at the completion of the procedure.

Following ERCP, the patient was managed with nasogastric decompression and intravenous carbapenem therapy. A repeat CT scan demonstrated no persistent duodenal mural defect. He remained clinically stable without evidence of ongoing leak or sepsis and was discharged home on 31 December 2025 after completing his course of antibiotics.

The patient re‐presented on 12 January 2026 with recurrent cholangitis and underwent repeat ERCP with biliary stent exchange. Follow‐up cross‐sectional imaging demonstrated complete resolution of the previously identified liver abscesses. He remains under hepatobiliary surgical follow‐up and is currently awaiting definitive CBD exploration.

## 3. Discussion

Endoscopic biliary drainage with plastic stent placement via ERCP is a widely used intervention for the management of biliary obstruction. Although stent migration is a recognised adverse event, the majority of migrated stents pass spontaneously through the gastrointestinal tract without causing symptoms. Perforation secondary to stent migration is uncommon and occurs in fewer than 1% of cases [1].

Several factors have been implicated in an increased risk of stent migration, including prolonged indwelling time (> 4 weeks), the use of large‐diameter stents (> 10F), benign biliary pathology, increased stent length (> 120 mm) and the use of straight plastic stents [5]. In contrast, the present case involved a shorter, curved stent and demonstrated unusually early migration, occurring within 10 days of insertion. The mechanism responsible for the early migration observed in this case remains uncertain. During the initial ERCP, the large CBD stone was intentionally left in situ because urgent biliary decompression was prioritised in the setting of severe ascending cholangitis with multiple liver abscesses. One possible explanation is that the retained stone exerted mechanical pressure on the proximal end of the stent during subsequent biliary decompression, facilitating distal displacement. Other contributing factors, including changes in bile duct calibre following drainage, papillary anatomy after sphincterotomy or patient‐specific anatomical characteristics, may also have influenced stent migration. Although these mechanisms cannot be confirmed, this case demonstrates that clinically significant migration may occur even when recognised risk factors are absent.

An important feature of this case is the successful endoscopic management of the perforation using a single through‐the‐scope clip. In previously published reports, many patients required multiple clips, over‐the‐scope devices or operative repair, particularly when diagnosis was delayed. Early recognition, together with the absence of generalised peritonitis, allowed definitive endoscopic treatment and avoided surgery.

Clinicians should maintain a high index of suspicion for stent‐related complications, even when initial recovery has been uneventful. Small retroperitoneal duodenal perforations may present with nonspecific or minimal symptoms, and delayed diagnosis has been associated with increased morbidity [4]. While abdominal radiography may demonstrate stent migration, CT remains the preferred imaging modality because it accurately identifies both the position of the migrated stent and associated perforation [1]. This case highlights the need for clinical vigilance in patients presenting with abdominal pain after biliary stent placement.

Management should be individualised according to the patient’s clinical condition and the characteristics of the perforation. Endoscopic closure is increasingly recognised as first‐line treatment for selected patients with contained duodenal perforations who remain haemodynamically stable and have no evidence of diffuse peritonitis [5]. Reported success rates for endoscopic closure of small duodenal defects (< 13 mm) using through‐the‐scope or over‐the‐scope clips range from 88% to 100%, with most patients avoiding operative intervention following successful closure [6].

The present case adds to the limited literature describing early duodenal perforation following migration of a short duodenal‐bend plastic biliary stent. Unlike most previously reported cases, perforation occurred within only 10 days of placement despite the absence of several recognised risk factors for stent migration and duodenal perforation. This observation is clinically important because it demonstrates that serious stent‐related complications may occur even in patients who would traditionally be considered at relatively low risk. Furthermore, successful closure was achieved using a single through‐the‐scope clip, avoiding surgical intervention. Together, these findings reinforce the need to maintain a high index of suspicion for stent‐related complications regardless of the presence or absence of established risk factors and support prompt endoscopic management as an effective treatment strategy in appropriately selected patients.

## 4. Conclusion

Duodenal perforation secondary to biliary stent migration is a rare but important complication that should be considered in any patients presenting with abdominal pain following ERCP, even shortly after stent placement. This case demonstrates that clinically significant perforation can occur despite the use of a shorter, curved stent and in the absence of recognised risk factors for stent migration and perforation. By highlighting a presentation outside of the conventional high‐risk profile, this case report extends current clinical knowledge and underscores the importance of maintaining clinical suspicion for stent‐related complications irrespective of perceived risk. Prompt recognition and early endoscopic intervention with clip closure in select cases can result in excellent outcomes and may mitigate the need for surgical management.

## Author Contributions

Shiraz Gabriel, Ahmed Abdelsalam and Desiree Moodley contributed to the clinical management of this patient. Renaldo Kortje provided surgical input. Tamzyn Huisamen drafted the manuscript. Desiree Moodley assisted with editing and revision.

## Funding

No specific funding was received for this work.

## Disclosure

All authors approved the final version.

## Consent

Written informed consent was obtained from the patient for publication of the case report and accompanying images.

## Conflicts of Interest

The authors declare no conflicts of interest.

## Supporting Information

Additional supporting information can be found online in the Supporting Information section.

## Supporting information


**Supporting Information** Timeline of clinical presentation, ERCP interventions and follow‐up.

## Data Availability

The data that support the findings of this study are available from the corresponding author upon reasonable request.
