# Supplementary material for: Early Duodenal Perforation Due to Migrated Plastic Biliary Stent, Successfully Managed Endoscopically: A Case Report
Source: Case Rep Gastrointest Med. 2026 Aug 20;2026:1965411. doi: 10.1155/crgm/1965411 (PMC13494346; doi:10.1155/crgm/1965411)
Supplement: Supplementary file 1 [file CRGM-2026-1965411-s001.docx]

**Supplementary Material:**

Timeline of Clinical Presentation, ERCP Interventions and Follow up

12 January 2026

Re-presented to hospital

- Recurrent cholangitis
- ERCP with biliary stent exchange

31 December 2025

Discharged home

17 December 2025

Repeat ERCP

- Distally migrated stent identified with subsequent duodenal perforation
- Perforation closed with TTS clip
- Cholangiogram showed patent biliary tree
- New plastic stent inserted with satisfactory biliary drainage

14 December 2025

- Re-presented with abdominal pain, nausea and vomiting
- CT suspicious for migrated biliary stent causing contained duodenal perforation

4 December 2025

Initial Presentation

- Cholangitis secondary to choledocolithiasis
- ERCP:

- 70-mm duodenal-bend plastic biliary stent inserted

- Biliary decompression achieved
